# Revealing the Effect of MnO_2_, Activated Carbon and MnO_2_/Activated Carbon on Chitosan Polymer Host Fabricated Co NPs: Antibacterial Performance and Degradation of Organic Compounds

**DOI:** 10.3390/polym14030627

**Published:** 2022-02-06

**Authors:** Hani S. H. Mohammed Ali, Sumiya, Yasir Anwar, Youssef O. Al-Ghamdi, Muhammad Fakieh, Shahid Ali Khan

**Affiliations:** 1 Department of Biological Sciences, Faculty of Science, King Abdulaziz University, P.O. Box 80203, Jeddah 21589, Saudi Arabia; haniolfat@gmail.com (H.S.H.M.A.); yasirpcsir2006@gmail.com (Y.A.); mf1881@yahoo.com (M.F.); 2 Princess Dr. Najla Bint Saud Al-Saud Center for Excellence Research in Biotechnology, King Abdulaziz University, P.O. Box 80203, Jeddah 21589, Saudi Arabia; 3 Center of Excellence for Advanced Materials Research (CEAMR), King Abdulaziz University, P.O. Box 80203, Jeddah 21589, Saudi Arabia; 4 Department of Chemistry, University of Swabi, Anbar, Swabi 23561, Pakistan; ssumiya208@gmail.com; 5 Department of Chemistry, School of Natural Sciences, National University of Science and Technology (NUST), Islamabad 44000, Pakistan; 6 Department of Chemistry, College of Science Al-Zulfi, Majmaah University, Al-Majmaah 11952, Saudi Arabia; Yo.alghamdi@mu.edu.sa

**Keywords:** chitosan, activated carbon, MnO_2_, Co NPs, antibacterial activity, degradation

## Abstract

MnO_2_ and MnO_2_ blended with 1 and 2 weight percent of activated carbon (AC), MnO_2_/AC1 and MnO_2_/AC2 were synthesized through the sol–gel method. The pure chitosan (CS) films were cast in the form of films. Similarly, 5 weight% of each MnO_2_, AC, MnO_2_/AC1 and MnO_2_/AC2 was intermingled with the CS to produce different films, such as CS-AC, CS-MnO_2_, CS-MnO_2_/AC1 and CS-MnO_2_/AC2. Zero-valent Co NPs were then supported on these films through the chemical reduction method and expressed as CS@Co, CS-AC@Co, CS-MnO_2_@Co, CS-MnO_2_/AC1@Co and CS-MnO_2_/AC2@Co NPs. All the catalysts were characterized by field emission scanning electron microscopy (FESEM), energy-dispersive spectroscopy (EDS) and X-ray diffraction (XRD) techniques. The synthesized catalysts were used as a dip catalyst against the hydrogenation of 4-nitrophenol (4NP), and for the degradation of methyl orange (MO) and Congo red (CR) dyes. The *k*_app_ and R^2^ values were deduced from pseudo-first-order kinetics for 4NP and MO and zero-order kinetics for CR dye. The *k*_app_ values of CS-AC@Co and CS-MnO_2_/AC1@Co NPs for 4NP hydrogenation were higher than those for any other member of the series, at 1.14 × 10^−1^ and 1.56 × 10^−1^ min^−1^ respectively. Similarly, the rate of CR degradation was highest with CS-AC@Co. The R^2^ values for 4NP, MO and CR dyes were above 0.9, which indicated that the application of pseudo-first- and zero-order models were appropriate for this study. Furthermore, the antibacterial activity of all the catalysts was evaluated against *Pseudomonas aeruginosa* and *Escherichia coli*. The CS-AC@Co NPs exhibited the highest zone of inhibition compared to other catalysts against *P. aeruginosa*, while all the catalysts were inactive against *E. coli*. This study reveals that the catalyst can be used for the degradation of other pollutants and for microbial inhibition.

## 1. Introduction

A dramatic increase in the anthropogenic activities, such as industrialization, modernization and urbanization, has contaminated water bodies to a serious extent. These activities are indeed exerting adverse effects on flora and fauna in ecosystems [1]. Nowadays, innumerable industries discharge their effluents directly into water bodies without any treatment, hence posing potential threats to aquatic biota [2]. The effluents from textile, paper, cosmetics, pharmaceuticals and food processing units are heavily loaded with dyes and other chemicals, which decrease the transparency of the water and stimulate their toxicity [3]. Dyes and dyestuff significantly block light penetration into water bodies, thereby impeding the vital activity of photosynthesis, hence rendering the entire ecosystem deprived of oxygen [4]. Reports have revealed that dyes and their degradation products are genotoxic and cytotoxic to living organisms [5,6]. Moreover, dyes accelerate immunological reactions, induce hyperactivity in children [7] and cause nasal congestion, urticaria, asthmatic disorders and rhinitis in men [8]. Besides the degradation of azo dyes, this study also aimed to decontaminate the water from hazardous chemicals such as 4-nitrophenol (4NP), which has been proven to be highly toxic to human beings. Indeed, 4NP is primarily discharged as an effluent by the pharmaceutical, pesticide, petrochemical and dye industries. Moreover, 4NP is a well-known potential carcinogen and mutagen; 4NP has been categorized as a priority contaminant by the United States (US) Environmental Protection Agency (EPA) [9]. As a consequence of insights into the hazardous effects caused by dyes and PNP, they should be removed from water bodies at the earliest opportunity.

In the last two decades, scientists and researchers have been attempting to remove as much dyestuff from water as possible [10]. In this regard, they have incorporated several water purification techniques, including adsorption, filtration [11] membrane technologies, Fenton’s oxidation processes, advanced oxidation processes, electrochemical oxidation, biological methods and the use of zero-valent metal (ZVM) nanocatalysis, which are documented in appropriate detail in the literature [12]. Biological methods for wastewater treatment represent the most promising techniques, owing to their simplicity, ease of applicability, versatility and low costs [13]. However, most of the synthetic dyes are non-biodegradable under their complex benzenoid frameworks; thus, biological treatments cannot be extended to a broader spectral domain of dyes [14]. Chemical treatment of wastewater, such as oxidation via ozone, ClO_2_ and chlorine, has also been proven to be very effective due to its remarkably high efficiency and reproducibility, but they generate chlorinated hydrocarbons as byproducts, which are well-established carcinogens [15]. Nowadays, advanced oxidation processes (AOPs) such as Fenton’s reagent [16], electro-Fenton’s, photo-electro Fenton’s, photocatalytic and photo-ozonation techniques, are the main areas of interest for environmental chemists worldwide for the decontamination of waste water [17], but their rising prices and operational difficulties limit the applicability of these techniques.

The introduction of catalytic degradation techniques for pollution control has gained the attention of researchers globally, to a greater extent than any other technique. Over the past few decades, among the catalytic techniques, the incorporation of zero-valent metal (ZVM) nanocatalytic techniques for water pollution abatement constitutes a newly emerging and highly promising tool due to its elevated catalytic potential [18,19,20,21,22]. The catalytic potential of ZVM and various metal oxide nanoparticles is attributed to their remarkably high surface to volume ratio, large surface energy and tiny quantum size effect, diversity, versatility and ease of application over a broad spectral regime of pollutants in water [23,24]. ZVM is synthesized in an eco-friendly way by treating a pristine or a supported metal ion with a suitable reducing agent such as NaBH_4_; however, ZVM NPs are always vulnerable to agglomeration and aggregation, which consequently leads to a loss of catalytic activity, but this problem can be easily avoided by using a suitable supporting matrix [25]. The supported catalyst after use can be conveniently recovered from the reaction mixture and can be reused as a dip catalyst. The employment of polymer-coated nanoscale composites for ZVM is gaining popularity in academic and industrial sectors due to their intrinsic features and significantly enhanced surface area. Some serious issues are always associated with the use of naked ZVM NPs for pollution abatement, such as the percolation of ZVM in the reaction mixture, and its cyclization, mass transport and effective isolation from the reaction mixture after use; however, these problems can be minimized by anchoring the ZVM over an appropriate polymeric support. Various polymer supports have been mentioned in the literature for the stabilization of zero-valent metal nanoparticles [10,26]. Chitosan is one of the emerging natural polymers that is used for NP stabilization due to their various inherent functional groups. Chitosan is a natural biopolymer with a linear structure and is derived from chitin. Chitosan possesses several important characteristics, such as biodegradability, non-toxic nature and biocompatibility; therefore, it is largely applied in various biomedical sectors [27,28]. All these characteristics make chitosan unique compared to other polymers; however, several drawbacks are associated with chitosan polymers, such as their low antimicrobial characteristics. To increase the antimicrobial characteristics and facilitate the anchoring of the NPs on their surface, several modifications have been adopted—for instance, the incorporation of inorganic fillers, which not only increases various characteristics of the polymer, but is also reported to increase the bioactivity of the polymer [1,29]. Therefore, the current study involved the stabilization of ZVM Co NPs over a variety of supports, such as pristine chitosan (CS), CS-blended composites with activated carbon (CS-AC), CS blended with MnO_2_ (CS-MnO_2_), CS-blended MnO_2_/AC1(CS-MnO_2_/AC1) and CS-blended MnO_2_/AC1(CS-MnO_2_/AC1). The above-mentioned composites were templates in the form of films and were employed as a solid supportive matrix for Co NPs’ stabilization; they were evaluated in terms of the inhibition of pathogenic bacteria. These stabilized Co NPs were utilized to address the degradation/reduction of target contaminants such as 4NP and different dyes to evaluate the comparative role of the matrix.

## 2. Experimental

### 2.1. Reagents and Materials

Chitosan polymer with 85% degree of acetylation was procured from BDH Company (London, England), and Congo red and methyl orange dyes, *p*-nitrophenol (4NP), NaBH_4_ and acetic acid were obtained from Dae-Jung Company (Sasang-gu, Busan, Korea). Moreover, CoCl_2_, MnCl_2_ salts and NaOH were purchased from Sigma Aldrich (Kawasaki, Kanagawa, Japan). Distilled water was obtained from the distillation plant of the Department of Chemistry, University of Swabi, KPK, Pakistan.

### 2.2. Instrumentation

The EDS and FESEM were performed on JSM-7600F, Tokyo, Japan, and FESEM JEOL (JSM-7600F, Japan). The X-ray diffraction (XRD) technique was performed on a PAN JDX-3532 JEOL Tokyo, JAPAN analytical diffractometer with a Cu Kα source of 1.5418 Å (40 kV, 30 mA, monochromatic). A UV–visible spectrophotometer was obtained from PerkinElmer Company (Lambda 365) and scanned from 190 to 800 nm (Waltham, MA, USA).

### 2.3. Synthesis of Materials and Composite Films

#### 2.3.1. Synthesis and Activation of Activated Carbon

The peanut shell was ground and heated at 400 °C for 6 h and then sieved with a 20 µm pore size sieve. After this, it was treated with concentrated HNO_3_, washed with distilled water and dried. The as-prepared activated carbon (AC) was used for the synthesis of other materials.

#### 2.3.2. Synthesis of MnO_2_

For the synthesis of MnO_2_ NPs, 1 M MnCl_2_ solution was prepared in 200 mL distilled water and basified with NaOH solution till pH 11, which was monitored on a pH meter. The solution was placed on a hot plate at 80 °C for 6 h. After the reaction’s completion, the supernatant was decanted and the precipitate was washed with a 7:3 ethanol–water mixture, and then the solution was centrifuged three times for the separation of the precipitate based on mass gravity. The precipitate was heated at 80 °C in an oven overnight and then we calcined the MnO_2_ NPs at 400 °C in a furnace for 6 h.

#### 2.3.3. Synthesis of MnO_2_/AC1 and MnO_2_/AC2

First, 1 M MnCl_2_ was mixed with 200 mL distilled water. After this, 1 and 2 weight% of AC was separately added to a beaker containing 100 mL of MnCl_2_ solution to prepare MnO_2_/AC1 and MnO_2_/AC2, respectively. Each solution was basified with a dilute solution of NaOH till pH 11, which was monitored by a pH meter. The mixture was placed on the hot plate at 80 °C for 6 h and then washed with ethanol and water mixture (7:3) and was centrifuged three times to separate the precipitate. The precipitate was dried at 80 °C overnight, and calcined at 400 °C for 6 h in a furnace. 

#### 2.3.4. Synthesis of Pure CS Films

The solution of CS was prepared by mixing 2 g CS in a 7:3 (*v*/*v*) acetic acid and water mixture. The mixture was stirred at room temperature until a clear paste formed. The CS paste was cast in a petri dish and uniformly dispersed in the form of sheets, and it was kept in an open environment. The solvent and water molecules were evaporated from the sheets; as a result, one face of the sheet was porous, while the other was smooth. The dried sheets were dipped in a concentrated solution of NaOH for a few minutes and then washed with distilled water to remove acid/base content. 

#### 2.3.5. Synthesis of CS Hybrid Sheets

Various sheets were prepared by blending the synthesized nanocomposite with CS host polymer. 

All the films, such as CS-AC, CS-MnO_2_, CS-MnO_2_/AC1 and CS-MnO_2_/AC2, were prepared by adding 5 weight% of AC, MnO_2_, MnO_2_/AC1 and MnO_2_/AC2, respectively, to the CS polymer and cast in a petri dish in the form of sheets. The remaining procedure for the sheet synthesis was the same as discussed above for the CS sheet preparation.

#### 2.3.6. Stabilization of Co NPs on CS and CS Hybrid Sheets

**Scheme 1:**
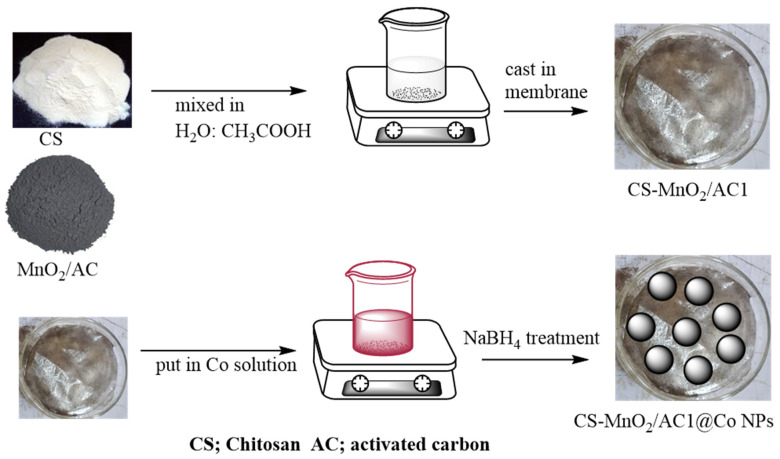
General depiction of the synthesis of CS-MnO_2_/AC1 and CS-MnO_2_/AC1@Co NPs.

The CS and CS hybrid sheets were dipped in 1 M Co salt solution for 6 h. All the sheets that adsorbed Co^2+^ ions were washed with distilled water to remove the un-adsorbed ions, and treated with freshly prepared NaBH_4_ solution, which changed the color of the sheets from pink to black. The black color was an indication of Co^0^ NPs synthesis. The synthesized Co NPs were used to treat discoloration caused by MO and CR dyes, as well as for the hydrogenation of 4NP. A generalized schematic representation is given in Scheme 1, including the preparation of CS-MnO_2_/AC1 and the synthesis of CS-MnO_2_/AC1@Co NPs.

### 2.4. Antibacterial Characteristics

The antibacterial potential of all the catalyst-supported Co NPs was assessed against *P. aeruginosa* and *E. Coli* on Mueller–Hinton agar using the Kirby–Bauer disk diffusion method [30]. The plates were sterilized, and then the culture of *P. aeruginosa* and *E. coli* was spread uniformly via a sterilized spreader across the whole plate. Different plates were used for *P. aeruginosa* and *E. coli*. After bacterial culture, the catalyst was cut into appropriate dimensions and then positioned in the plates containing bacterial colonies. These plates were incubated at 37 °C for 24 h and the zone of inhibition was measured through a scale in cm. The zone of inhibition was calculated through the mean value around the nutrient agar disk. 

### 2.5. Evaluation of Catalyst Activity in 4NP Reduction and Dye Degradation

All the catalysts’ activities were assessed for the hydrogenation of 4NP and degradation of MO and CR dyes. Briefly, 0.2 mM 4NP solution was prepared in 100 mL of distilled water and then 2.5 mL of it was placed in a cuvette and the absorption noted. After this, 0.5 mL of NaBH_4_ (0.5 mM) was added to the same cuvette and then 20 mg of the catalyst was added. The hydrogenation of 4NP or degradation of dyes was recorded using a UV–vis spectrophotometer. Similarly, 0.1 mM solutions of CR and MO were prepared in 100 mL of distilled water and then 2.5 mL of each dye was placed in a cuvette, along with 0.5 mL NaBH_4_ (0.5 mM) solution and 20 mg of the catalyst, and their absorbance was recorded on the UV–vis spectrophotometer (Waltham, MA, USA).

## 3. Results and Discussion

### 3.1. FESEM and EDS

**Figure 1:**
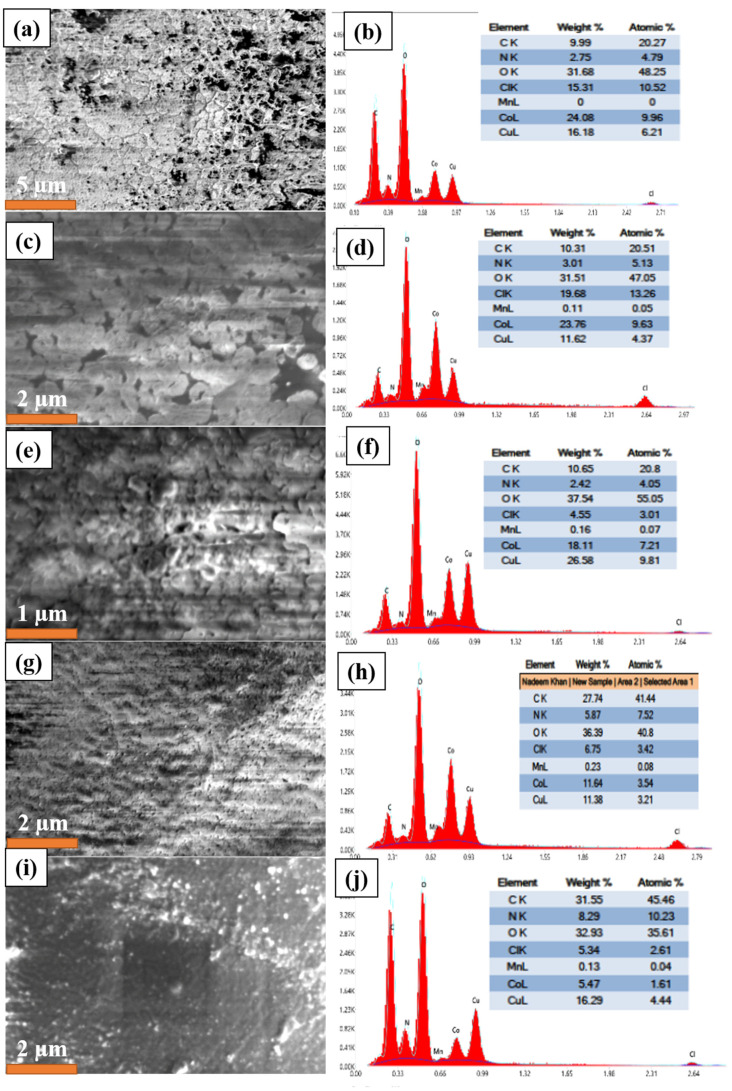
The FESEM and EDS images of CS@Co NPs (**a**,**b**), CS-AC@Co NPs (**c**,**d**), CS-MnO_2_@Co NPs, (**e**,**f**), CS-MnO_2_/AC1@Co NPs (**g**,**h**) and CS-MnO_2_/AC2@Co NPs (**i**,**j**).

The FESEM images and EDS spectrum are provided in the inset of Figure 1. The left- and right-hand sides of Figure 1 present the FESEM images and EDS spectrum. The FESEM image of CS@Co NPs shows a smooth surface with small pores (Figure 1a). The EDS spectrum and elemental window indicate peaks for C, O and N with 9.99, 31.68 and 2.75 weight%. The C, O and N atoms arise from the chitosan skeleton. Other peaks arise for Co and Cu elements. The Co and Cu elements are present in 24.08 and 16.18 by weight% (Figure 1b). Peaks for Cu are observed throughout the EDS spectrum, which is due to the Cu sputtering. Cu sputtering was performed before the FESEM and EDS analyses. Figure 1c indicates that AC covered the CS polymer and there were voids in their morphology. The EDS spectrum indicated C, O, N and Co elements with 10.31, 31.51, 3.01 and 23.76 weight percent in the CS-AC@Co catalyst (Figure 1d). Similarly, the FESEM spectrum of CS-MnO_2_ indicated a rough surface of the polymer sheet, with small sphere-shaped embedded Co NPs (Figure 1e). The elemental window indicated peaks for C, O, N, Mn and Co elements, which were present in 10.65, 37.54, 2.42, 0.16 and 18.11 by weight percent in the CS-MnO_2_@Co NPs (Figure 1f). The FESEM image of CS-MnO_2_/AC1 (Figure 1g) indicated a protruding surface, while the EDS indicated peaks for C, O, N and Co elements with 27.74, 36.39, 5.87, 0.23 and 11.64 weight percent in the CS-MnO_2_/AC1@Co catalyst (Figure 1h). The CS-MnO_2_/AC2@Co catalyst showed a flat surface of the films, with numerous white spots on them (Figure 1i). The EDS and elemental window indicated peaks for C, N, O, Mn and Co with 31.55, 8.29, 32.93, 0.13 and 5.47 weight%, respectively (Figure 1j).

### 3.2. XRD

**Figure 2:**
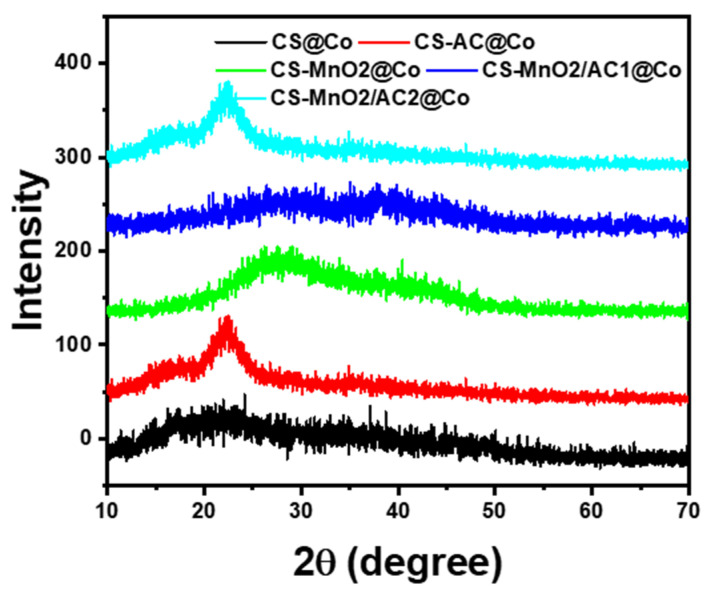
XRD spectrum of the synthesized catalysts.

The XRD spectrum of the synthesized catalysts is shown in Figure 2. The XRD spectra of CS@Co, CS-AC@Co, CS-MnO_2_/AC1@Co and CS-MnO_2_/AC2@CoNPs indicated an amorphous peak at 2θ = 22.5°, while CS-MnO_2_@Co exhibited an amorphous peak at 27.8°. These amorphous peaks reveal that Co NPs grew in an amorphous nature during their fabrication. The literature also includes similar reports for Co and Cu NPs, respectively [31,32]. Therefore, it is suggested that Co NPs with a larger particle size are formed on CS and CS hybrid catalysts during their synthesis. 

### 3.3. Antibacterial Activity

**Figure 3:**
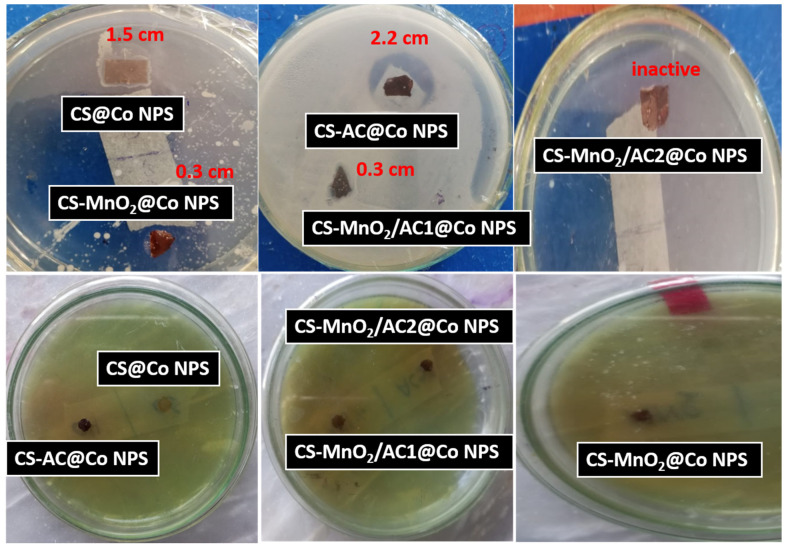
Antibacterial activity of all the catalysts against *P. aeruginosa* (**upper row**) and *E. coli* (**lower row**).

Antibacterial characteristics are important for applications in the biomedical field [33,34]. All the catalysts were tested against two bacterial species, *P. aeruginosa* and *E. coli*, for 24 h of incubation time. The upper row of Figure 3 presents the disk diffusion results for *P. aeruginosa*, while the lower row show the results for *E. coli*. The zones of inhibition of all the catalysts were measured in cm. In the current study, CS-AC@Co NPs exhibited the strongest antibacterial activity, inhibiting a 2.2 ± 0.1 cm zone of *P. aeruginosa*, while the CS@Co NPs showed a 1.5 ± 0.1 cm zone of inhibition. Similarly, CS-MnO_2_@Co and CS-MnO_2_/AC1@Co NPs also showed an approximately 0.3 ± 0.1 cm zone of inhibition, while CS-MnO_2_/AC2@Co NPs was inactive against *P. aeruginosa*. Furthermore, all the synthesized catalysts was inactive against *E. coli*.

### 3.4. Catalyst Activity

#### 3.4.1. Hydrogenation of 4NP

The hydrogenation of the –NO_2_ group of 4NP was carried out by using NaBH_4_ as a reductant to assess the catalyst activity of CS@Co, CS-AC@Co, CS-MnO_2_@Co, CS-MnO_2_/AC1@Co and CS-MnO_2_/AC2@Co NPs. Firstly, 4NP exhibited a peak at 315 nm in the absorbance spectrum, with a light yellow color; however, soon after the addition of NaBH_4_, a red shift was observed and the λ_max_ changed to 400 nm, with a deep yellow color [35]. The increase in the wavelength of 4NP was due to the increase in conjugation in the nitrophenolate anion. Numerous studies are available indicating that a reducing agent has a negligible effect on the degradation of 4NP, and thus the hydrogenation of the –NO_2_ group in the presence of a reducing agent is considered a kinetically unfavorable reaction [36,37,38]. However, many catalyst systems have been described by various researchers for the hydrogenation of the –NO_2_ group [1,39,40,41]; nevertheless, a more efficient and retrievable catalyst system is required. The hydrogenation of 4NP is considered an atom economic reaction where 4-aminophenol (4AmP) is the only major product. Furthermore, this reaction can be easily recorded on a UV–vis spectrophotometer. The decrease in the absorbance of 4-nitrophenolate was recorded at 400 nm as the reaction progressed, where a peak at 290 nm was attributed to the formation of the 4AmP product. 

**Figure 4:**
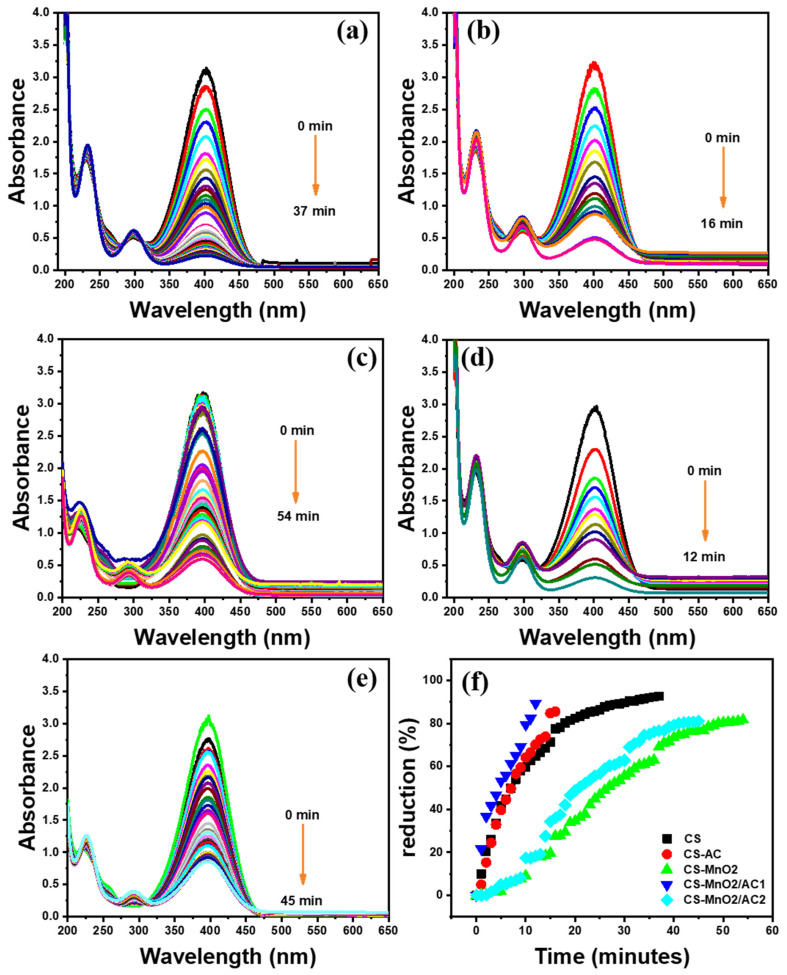
Absorbance spectrum of 4NP reduction in the presence of NaBH_4_ by using CS@Co (**a**), CS-AC@Co (**b**), CS-MnO_2_@Co (**c**), CS-MnO_2_/AC1@Co (**d**), CS-MnO_2_/AC2@Co NPs (**e**) and percentage reduction as a function of time (**f**). Experimental conditions: 2.5 mL of 0.2 mM 4NP solution; 0.5 mL of 0.5 mM NaBH_4_ solution and 20 mg of each catalyst.

The decline in the absorbance at 400 nm was documented after the addition of CS@Co, CS-AC@Co, CS-MnO_2_@Co, CS-MnO_2_/AC1@Co and CS-MnO_2_/AC2@Co NPs. As depicted in Figure 4a, the CS@Co NPs reduced 92.62% of 4NP to 4AmP in 37 min, 81.67% by CS-AC@Co in 16 min (Figure 4b), 85.55% by CS-MnO_2_@Co in 54 min (Figure 4c) and 89.63% by CS-MnO_2_/AC1@Co in 12 min (Figure 4d), while CS-MnO_2_/AC2@Co NPs reduces 81.60% of 4NP to 4AmP in 45 min (Figure 4e). The percent reduction of the –NO_2_ group of 4NP to –NH_2_ of 4AmP is presented in Figure 4f. The rate constant data were extracted from the pseudo-first-order kinetics, which indicated the highest value for CS-MnO_2_/AC1@Co NPs, which was 1.56 × 10^−1^ min^−1^, and the slowest for CS-MnO_2_@Co NPs. The rate constant value and the % reduction of the –NO_2_ group to –NH_2_ indicated that the addition of either AC or MnO_2_/AC composite had a significant effect on the chitosan polymer and enhanced the rate of this hydrogenation reaction. However, amongst all the catalysts, the CS-MnO_2_/AC1@Co NPs showed superior catalyst performance compared to other catalysts in this study. However, as is obvious from Table 1, all the hybrid films indicated stronger activity as compared to the CS, except CS-MnO_2_@Co NPs; therefore, we can conclude that the addition of AC or MnO_2_/AC played a prominent role in the improvement of the CS polymer host. Furthermore, the value of R^2^ indicates that the experimental data are in good agreement and the pseudo-first-order kinetic model is appropriate. 

#### 3.4.2. Discoloration of MO Dye

**Figure 5:**
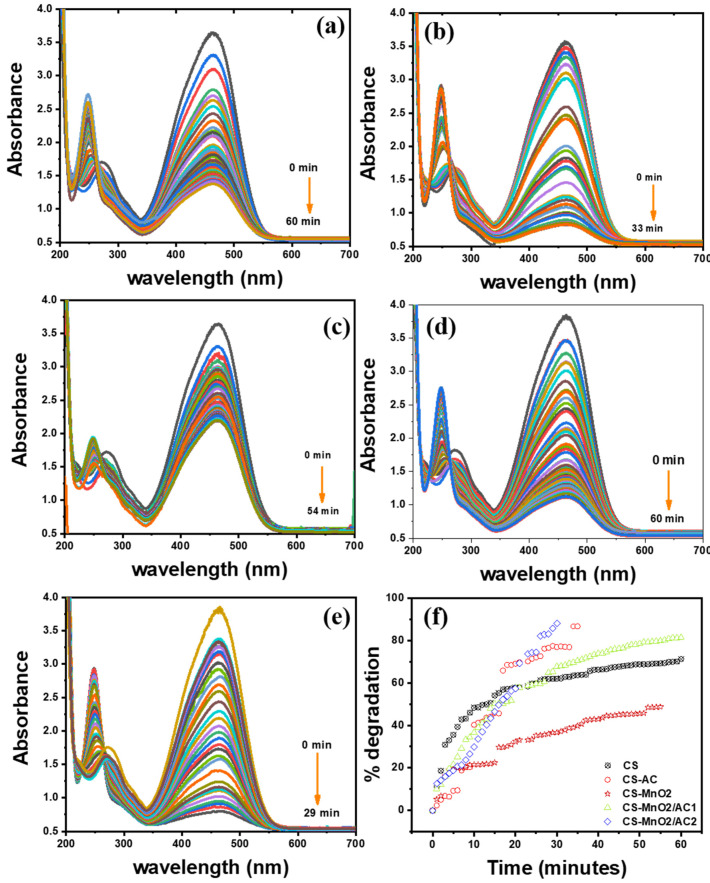
Absorbance spectrum of MO degradation in the presence of NaBH_4_ by using CS@Co (**a**), CS-AC@Co (**b**), CS-MnO_2_@Co (**c**), CS-MnO_2_/AC1@Co (**d**) and CS-MnO_2_/AC2@Co NPs (**e**) and % degradation of MO dye (**f**). Experimental conditions: 2.5 mL of 0.1 mM MO solution; 0.5 mL of 0.5 mM NaBH_4_ solution and 20 mg of each catalyst.

All the catalysts were applied to treat the discoloration caused by MO dye in the presence of NaBH_4_. MO dye is a mono azo dye with a –N = N– functional group. A peak at 465 nm appeared in the absorbance spectrum of the MO dye; however, the azo group of MO dye reduces to the –NH_2_-NH_2_– group after the addition of NaBH_4_. It is noted in the literature that the MO dye is not or is less degraded in the presence of NaBH_4_, and thus it is kinetically an unfavorable process. The degradation of MO dye at 465 nm was recorded using all the catalysts in the presence of NaBH_4_; as a result, a peak at 250 nm was observed. The absorbance spectrum of CS@Co NPs is presented in Figure 5a, which shows that approximately 71.19% of the MO dye was decolorized in 60 min. The CS-AC@Co NPs degraded 86.64% of MO dye in 33 min (Figure 5b), while CS-MnO_2_@Co NPs degraded only 48.61% in 54 min (Figure 5c). Similarly, CS-MnO_2_/AC1 and CS-MnO_2_/AC2 decolorized 81.42 and 87.97% of MO dye in 60 and 29 min (Figure 5d,f), respectively. The % degradation of MO dye with all the catalysts is presented in Figure 5f. Based on the data, it is inferred that MnO_2_ further diminished the catalyst activity of CS, while the AC, MnO_2_/AC1 and MnO_2_/AC2 enhanced the catalyst activity compared to CS. The pseudo-first-order model was used to assess the kinetic rate of all the catalysts against the degradation of MO dye. The experimental data fitted well in the pseudo-first-order model; therefore, this model was applied to MO degradation to deduce the *k*_app_ and R^2^ values. As manifested in Table 1, the highest catalyst activity was displayed by CS-MnO_2_/AC2, with a rate constant value of 6.75 × 10^−2^ min^−1^, followed by CS-AC@Co NPs (5.81 × 10^−2^ min^−1^), while the lowest MO degradation rate was displayed by CS-MnO_2_@Co NPs (1.05 × 10^−2^ min^−1^). The linearity of all the catalysts except CS@Co NPs was above 0.9, which indicates that the experimental data fitted well in the pseudo-first-order model. 

#### 3.4.3. Discoloration of CR Dye

**Figure 6:**
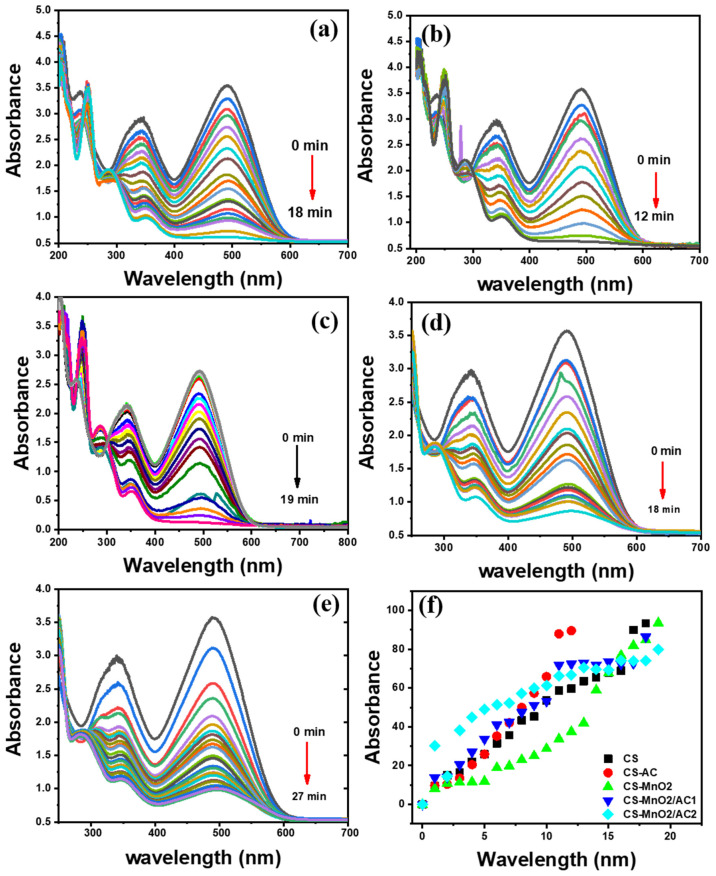
Absorbance spectra of CR degradation in the presence of NaBH_4_ by using CS@Co (**a**), CS-AC@Co (**b**), CS-MnO_2_@Co (**c**), CS-MnO_2_/AC1@Co (**d**) and CS-MnO_2_/AC2@Co NPs (**e**), and % degradation of CR dye (**f**). Experimental conditions: 2.5 mL of 0.1 mM CR solution; 0.5 mL of 0.5 mM NaBH_4_ solution and 20 mg of each catalyst.

CR is a diazo dye and its λ_max_ appeared at 495 nm in the UV–vis spectrum. The azo group is converted to the hydrazine group after NaBH_4_ treatment [38,42]. As with 4NP and MO dye, the CR dye is also kinetically an unfavorable process with NaBH_4_ treatment [40,43]. A decrease in the absorbance of the CR dye at 495 nm was recorded during the UV–vis spectroscopy and a few new peaks were noted at 248 and 280 nm due to the formation of amine-containing products. The CS@Co NPs degraded 93.28% of CR dye (see Figure 6a for the absorbance spectrum) in 18 min. Similarly, CS-AC@Co NPs (Figure 6b) decolorized 89.49% of CR dye in 12 min, while CS-MnO_2_@Co NPs (Figure 6c) decolorized 93.53% in 19 min. The CS-MnO_2_/AC1@Co (Figure 6d) and CS-MnO_2_/AC2@Co NPs (Figure 6e) decolorized 86.31% and 83.44% CR dye in 18 and 27 min, respectively. The % discoloration of CR dye is depicted in Figure 6f. Unlike the 4NP and MO dye, the experimental data of the CR dye are fixed in the zero-order kinetics because the linearity of the zero-order kinetics for CR degradation is greater than that of the pseudo-first-order kinetics model. The rate constant with CS-AC@Co NPs was the highest, at 2.25 × 10^−1^ min^−1^, and the lowest was observed with CS-MnO_2_/AC2, at 6.75 × 10^−2^ min^−1^. The R^2^ values of all the catalysts except CS-MnO_2_/AC2 were above 0.9, which suggests the agreement of the data in the zero-order kinetics (Table 1).

#### 3.4.4. Recyclability of the Catalyst

**Figure 7:**
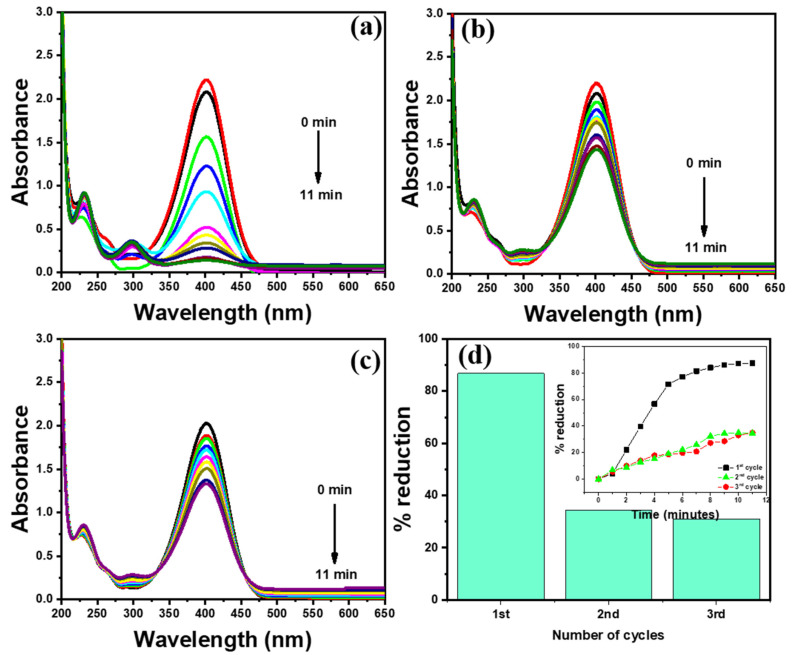
Recyclability of the CS-AC catalyst against 4NP reduction. The absorbance spectrum of 4NP reduction for the 1st cycle (**a**), 2nd cycle (**b**) and 3rd cycle (**c**), and the % reduction of 4NP against the number of cycles (**d**). The bar graph shows the % reduction of 4NP as a function of time in Figure 7d. Experimental conditions: 4NP (2.5 mL of 0.13 mM); NaBH_4_ (0.5 mL of 0.5 mM); 20 mg of CS-AC catalyst.

The recyclability of the CS-AC catalyst was evaluated against the hydrogenation reaction of the 4NP reaction. First, 0.13 mM solution of the 4NP was treated with 0.5 mM solution of NaBH_4_ and the reaction’s progress was recorded using the UV–vis spectrophotometer. The reaction was completed in 11 min, and afterwards, the catalyst was fed to the second cycle for 11 min and similarly for the third cycle under the same experimental conditions. As shown in Figure 7a, the CS-AC@Co NPs reduced approximately 85% of 4NP to AmP in 11 min; however, the second and third (see Figure 7b,c for UV–vis spectrum) cycles approximately reduced 40% of 4NP to 4AmP in 11 min. As is clear from Figure 7, the catalyst activity was lost after the first cycle; however, no further loss in activity was observed in the second and third cycles. This can be explained by the low availability of the catalyst’s active sites after the first cycle. Figure 7d presents a bar graph of the % reduction of 4NP and the inset in Figure 7d shows the % reduction of 4NP as a function of reaction time. 

## 4. Conclusions

In the current study, various nanocomposites, including MnO_2_, MnO_2_/AC1 and MnO_2_/AC2, were synthesized through the sol–gel process. The effect of these nanocomposites along with activated carbon (AC) was revealed on chitosan polymer (CS), and 5 weight percent of each nanocomposite and AC was added as a filler in the CS polymer and cast in the form of films. All these films were fabricated with Co^+2^ ions and then converted to their corresponding Co NPs by treatment with NaBH_4_. Among all the catalysts, CS-AC@Co NPs indicated good antibacterial activity against *P. aeruginosa*, while all the catalysts were inactive against *E. coli*. The synthesized Co NPs on the CS and CS hybrid film templates were used as a dip catalyst for the hydrogenation of 4NP and degradation of MO and CR dyes. The rate constant value of 4NP and MO dye was extracted from the pseudo-first-order model, while it was based on zero-order kinetics for CR dye degradation. The rate constant value for 4NP hydrogenation was higher for CS-MnO_2_/AC1@Co and CS-AC@Co NPs (1.56 × 10^−1^ and 1.14 × 10^−1^ min^−1^, respectively). Furthermore, the degradation of MO and CR dye was also well executed using CS-AC@Co, CS-MnO_2_/AC1@Co and CS-MnO_2_/AC1@Co NPs. The CS-AC@Co NPs also showed good recyclability for the hydrogenation of 4NP. In the first cycle, approximately 84% of 4NP was reduced to 4AmP in 11 min, which was reduced to 38% in the second and third cycles, respectively. However, a negligible loss of catalyst activity was observed in the second and third cycles.

This work applies generally to the removal of azo dyes and reduction of 4NP, as well as the inhibition/killing of *P. aeruginosa* and *E. coli.* However, these catalysts may be used for the degradation of different dyes, reduction of nitroaromatic compounds and inhibition/killing of different microbes. 

## Figures and Tables

**Table 1:** Rate constant, R^2^ values and % reduction or degradation values of 4NP and MO and CR dyes by using Co NPs supported on different polymer sheets.

Catalyst	Analyte	R^2^	*k*_app_	% Degradation
CS@Co	4NP	0.9835	7.01 × 10^−2^	92.62
CS-AC@Co		0.9698	1.14 × 10^−1^	81.67
CS-MnO_2_@Co		0.9821	3.59 × 10^−2^	85.55
CS-MnO_2_/AC1@Co		0.9371	1.56 × 10^−1^	89.63
CS-MnO_2_/AC2@Co		0.9842	4.28 × 10^−2^	81.60
CS@Co	CR	0.9792	1.43 × 10^−1^	93.28
CS-AC@Co		0.9622	2.25 × 10^−1^	89.49
CS-MnO_2_@Co		0.9171	1.44 × 10^−1^	93.53
CS-MnO_2_/AC1@Co		0.9410	1.33 × 10^−1^	86.31
CS-MnO_2_/AC2@Co		0.7940	6.45 × 10^−2^	83.44
CS@Co	MO	0.8543	1.50 × 10^−2^	71.19
CS-AC@Co		0.9558	5.81 × 10^−2^	86.64
CS-MnO_2_@Co		0.9543	1.05 × 10^−2^	48.61
CS-MnO_2_/AC1@Co		0.9977	4.36 × 10^−2^	81.42
CS-MnO_2_/AC2@Co		0.9119	6.75 × 10^−2^	87.97

The *k*_app_ and R^2^ values of 4NP and MO are based on pseudo-first-order kinetics, while the *k*_app_ and R^2^ values for CR discoloration were deduced from zero-order kinetics.

## Data Availability

The data presented in this study are available on request from the corresponding author.
